# Fast nanoscale imaging of strain in a multi-segment heterostructured nanowire with 2D Bragg ptychography

**DOI:** 10.1107/S1600576723010403

**Published:** 2024-02-01

**Authors:** Susanna Hammarberg, Dmitry Dzhigaev, Lucas A. B. Marçal, Vilgailė Dagytė, Alexander Björling, Magnus T. Borgström, Jesper Wallentin

**Affiliations:** a Synchrotron Radiation Research and NanoLund, Lund University, Box 118, Lund 22100, Sweden; b MAX IV Laboratory, Lund University, Lund 22100, Sweden; c Solid State Physics and NanoLund, Lund University, Box 118, Lund 22100, Sweden; Montanuniversität Leoben, Austria

**Keywords:** Bragg projection ptychography, III–V materials, nanowires, X-ray imaging

## Abstract

Developing semiconductor devices requires a fast and reliable source of strain information with high spatial resolution and strain sensitivity. This work investigates the strain in an axially heterostructured 180 nm-diameter GaInP nanowire with InP segments of varying lengths down to 9 nm, simultaneously probing both materials. Scanning X-ray diffraction (XRD) is compared with Bragg projection ptychography (BPP), a fast single-projection method. BPP offers a sufficient spatial resolution to reveal fine details within the largest segments, unlike scanning XRD. The spatial resolution affects the quantitative accuracy of the strain maps, where BPP shows much-improved agreement with an elastic 3D finite element model compared with scanning XRD. The sensitivity of BPP to small deviations from the Bragg condition is systematically investigated. The experimental confirmation of the model suggests that the large lattice mismatch of 1.52% is accommodated without defects.

## Abstract 2

A strained heterostructured nanowire is investigated using 2D Bragg ptychography and scanning X-ray diffraction.

## 1. Introduction

The ever-increasing range of applications of semiconductor nanostructures stems from their advantages in terms of scalability, electronic tunability, material reduction and price. Changes in physical properties at the nanoscale can be used to tailor the performance of semiconductor nanostructure devices. Semiconductor nanowires are attractive constituents for electronic devices in many applications, including energy-harvesting photovoltaics (Haverkort *et al.*, 2018 ▸; Otnes & Borgström, 2017 ▸), light-emitting diodes (Gudiksen *et al.*, 2002 ▸; Gibson *et al.*, 2019 ▸; Barrigón *et al.*, 2019 ▸; Motohisa *et al.*, 2019 ▸) and electronics (Memisevic *et al.*, 2017 ▸; Tomioka *et al.*, 2012 ▸; Jia *et al.*, 2019 ▸).

The energy bands in semiconductors can be engineered by creating heterostructures, which are commonly used to build semiconductor lasers and solar cells. Creating a nanowire heterostructured in the axial direction involves changing the band gap along the transport direction, tailoring the energy landscape for the moving charges. Nanowire heterostructures are being explored for devices such as multijunction solar cells (LaPierre *et al.*, 2013 ▸; Yao *et al.*, 2015 ▸), tunnel diodes (Zeng *et al.*, 2018 ▸), tunnel field-effect transistors (Memisevic *et al.*, 2017 ▸) and lasers (Saxena *et al.*, 2013 ▸). We are studying the Ga_*x*_In_1−*x*_P semiconductor as a promising material for photovoltaics (Hrachowina *et al.*, 2022 ▸) as its bandgap can be tuned from the near-infrared region to the middle of the visible spectrum.

In the nanowire format, theoretical calculations have predicted that higher lattice mismatches without defect formation can be reached than in the bulk (Ertekin *et al.*, 2005 ▸) so that ordinarily incompatible material combinations can be feasible. However, the theory is based on several simplifications that ignore phenomena such as bending (Wallentin *et al.*, 2017 ▸) and interdiffusion, highlighting the need for experimental studies.

The design of the nanowire transport properties benefits from complete and precise feedback on the crystal structure and variations therein, including strain and bending. Strain is related to displacements in the crystal lattice and has a close relationship to bending, which tilts the crystal lattice. Nanowire heterostructures usually have an innate strain from lattice mismatch which, in a device, can be further affected by processing such as metal contacts (Lazarev *et al.*, 2018 ▸). These variations in the crystal affect the band gap on a length scale that can influence the device performance (Hrachowina *et al.*, 2022 ▸). Regardless of whether the lattice deformations originate from conscious design to tune the performance or unwanted effects spawned from growing or processing, it is vital to be able to measure the full strain distribution in the nanowires.

As the dimensions of semiconductor-based devices decrease to the nanoscale, there are, in addition to the necessity of high strain sensitivity, increasingly higher demands on the spatial resolution of the methods for probing their structure. Transmission electron microscopy enables the characterization of nanostructures with atomic resolution (Hÿtch & Minor, 2014 ▸); however, the strain sensitivity is typically modest. X-rays offer superior strain sensitivity, and their high penetration depth circumvents the need for destructive sample preparation for embedded structures, as is often the case for electron-based probes.

The diffracted signal from a nanocrystal, illuminated with a nano-focused X-ray beam, is sensitive to distortions in the crystal lattice. With scanning X-ray diffraction (XRD), crystal strain can be mapped by analyzing shifts of the diffraction patterns in reciprocal space (Marçal *et al.*, 2015 ▸). Scanning XRD has been used to characterize nanowire devices and heterostructures (Spolenak *et al.*, 2003 ▸ Stankevič *et al.*, 2015 ▸; Wallentin *et al.*, 2016 ▸; Krause *et al.*, 2016 ▸; Schülli & Leake, 2018 ▸; Marçal *et al.*, 2020 ▸; Dzhigaev *et al.*, 2020 ▸). With this method, the spatial resolution is limited by the beam size and hence restrained by the capacity of the X-ray focusing optics. In regions with variations in strain, the limited spatial resolution leads to averaging and underestimation of real local strain maxima.

In order to overcome the spatial resolution limit, coherent diffractive imaging (CDI) methods have been developed to give sub-beam resolution by means of oversampling diffraction patterns measured with a coherent X-ray beam (Sayre, 1952 ▸). The measured diffraction pattern is the modulus squared of the scattered wave, but phase-retrieval algorithms can numerically reconstruct the missing phases (Fienup, 1982 ▸), as first experimentally demonstrated in forward-scattering geometry (Miao *et al.*, 1999 ▸). For a Bragg peak that is extended in reciprocal space, the condition for phase retrieval is oversampling with twice the Nyquist sampling rate (Sayre, 1952 ▸). The adaptation Bragg coherent diffraction imaging (BCDI) encodes the crystal strain in the phase of the reconstructed object and is capable of imaging crystal deformations of nanocrystals in three dimensions. BCDI reconstructions have been demonstrated on various materials (Pfeifer *et al.*, 2006 ▸; Shapiro *et al.*, 2005 ▸; Robinson & Harder, 2009 ▸; Newton *et al.*, 2010 ▸) with 3D resolution on the nanoscale. There are examples of BCDI on nanowires (Diaz *et al.*, 2009 ▸; Lazarev *et al.*, 2018 ▸; Dzhigaev *et al.*, 2016 ▸), but in general, the oversampling conditions for BCDI are not suitable for extended objects such as nanowires. Although the object should be fully enclosed by the coherence volume of the beam, there is a contradicting demand for high flux, which requires a focused beam as the scattering is weak.

To enable coherent imaging of extended objects, CDI was combined with standard scanning microscopy methods with the development of ptychography, first in forward-scattering geometry (Rodenburg *et al.*, 2007 ▸; Thibault *et al.*, 2008 ▸) and later in Bragg geometry (Godard *et al.*, 2011 ▸; Huang *et al.*, 2012 ▸). Here, the object is scanned with overlapping adjacent steps of the beam, ensuring redundancy of the diffracted signal. At each scanning point, 3D diffraction patterns are collected by sampling the Bragg peak in two dimensions on an area detector and in the third by performing a rocking curve, slightly rotating the object at angles close to the Bragg condition. This has enabled imaging of extended nano­structures spatially resolved in three dimensions with nano-resolution and strain sensitivity of 10^−4^ (Berenguer *et al.*, 2013 ▸; Pateras *et al.*, 2015 ▸; Chamard *et al.*, 2015 ▸; Li *et al.*, 2021 ▸, 2022 ▸). However, collecting the 3D diffraction patterns in Bragg ptychography poses a serious challenge, as the positions of the beam in real space need to match at every step along the rocking curve. Furthermore, 3D Bragg ptychography is time consuming and the high dose can lead to beam damage.

To overcome these limitations, alternative approaches where the need for the whole rocking curve is redundant have been developed, including single- and multi-angle Bragg projection ptychography (BPP) in two (Hruszkewycz *et al.*, 2012 ▸; Dzhigaev *et al.*, 2017 ▸; Holt *et al.*, 2014 ▸) and three dimensions (Hill *et al.*, 2018 ▸; Hruszkewycz *et al.*, 2017 ▸). In particular, BPP is based on a single angle, which leads to much shorter measurement times and less stringent mechanical demands. BPP can reconstruct the projected phase, which is proportional to the projected displacement field.

**Figure 1:**
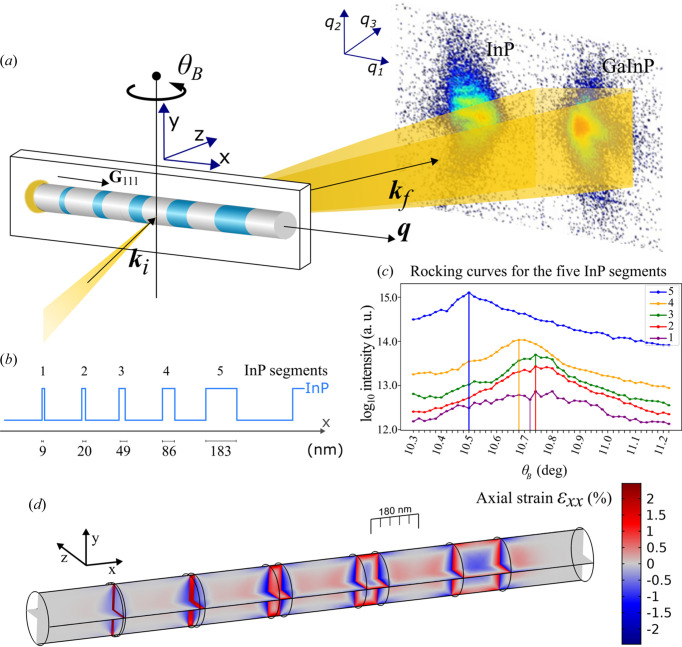
(*a*) Sketch of the BPP experiment. The heterostructured nanowire lies flat on a substrate and is placed horizontally in the beam with the gold growth seed particle to the left. The nanowire is in the Bragg condition for the {111} lattice planes, which are orthogonal to the nanowire axis. Thus, the scattering vector **q** is parallel with the nanowire axis. The nanowire is translated in the nanofocused beam along *x* and *y*, whereas the scattered signal is measured with a 2D detector in the far field in the measurement coordinate system (*q*_1_, *q*_2_, *q*_3_). The *x* and *y* translation is repeated at different rotations in θ_B_. (*b*) Lineout of the nanowire showing the nominal lengths of the five InP segments in the GaInP nanowire. The segments are denoted with numbers from left to right, segments 1–5. (*c*) Summed intensity as a function of rotation θ_B_ (rocking curves) from the five respective InP segments in logscale. The vertical lines indicate the Bragg angles 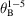 , chosen from the maximum of Gaussian fits to the peaks, except for 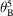 where the maximum intensity point was chosen. (*d*) 2D cuts of axial strain 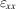 from a 3D FEM model. Positive values of 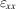 correspond to tensile strain in the axial direction.

The averaging along the scattered beam is rather unproblematic for samples that are homogeneous along this direction, but generally, samples will have strain that varies in all directions. In this work, we have investigated axial strain in a Ga_*x*_In_1−*x*_P/InP heterostructure nanowire with five InP segments ranging from 180 to 8 nm in length. At the nominal lattice mismatch of 1.52%, the radius of 90 nm is beyond the calculated critical radius (Ertekin *et al.*, 2005 ▸), which is the threshold below which the lattice mismatch can be accommodated with expansion or compression without forming defects. As shown in Fig. 1 ▸(*d*), simulations based on finite element modeling (FEM) show complex 3D strain distributions with sharp gradients. In the shorter segments, the strain is relatively homogeneous, whereas the larger ones have large internal variations. Thus, this is a challenging sample for any strain-mapping method.

Here, we compare measurements of displacement fields and strain within the InP segments using conventional scanning XRD with those using BPP, as well as with simulated experiments based on FEM. Comparisons are made of both the phase, which is proportional to the displacement field, and the gradient thereof, *i.e.* the strain. We find that the limited spatial resolution of scanning XRD makes this method unable to resolve variations within the segments and that it leads to quantitatively incorrect results in both larger and smaller segments. In contrast, BPP resolves fine details within the segments with reasonable quantitative agreement with simulations for the shorter segments. Furthermore, we show how the Ga_*x*_In_1−*x*_P and InP segments can be probed simultaneously, producing sub-beam resolved strain maps for the complete nanowire heterostructure in one measurement.

## 2. Materials and methods

### 2.1. Theory

The scattering geometry is depicted in Fig. 1 ▸(*a*). The scattered intensity is measured as a function of the scattering vector **q** = **k**_f_ − **k**_i_, where **k**_i_ and **k**_f_ represent the incoming and outgoing wavevectors, respectively. The Laue condition is fulfilled when **q** = **G**_*hkl*_, where **G**_*hkl*_ is the reciprocal lattice vector specified with the Miller indices *hkl*. If *q* is measured at the Laue condition, this probes the *d*-spacing planes specified with *hkl* since

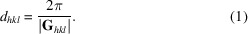

The far-field diffraction pattern from a nanocrystal illuminated with a coherent X-ray beam encodes the displacement field **u**(**r**) (where **r** is a real space vector) in the crystal, describing the displacements from an ideal lattice. To describe how **u**(**r**) is probed in Bragg ptychography, we start by introducing a complex density representing the object:

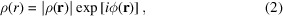

where 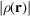 is the electron density, and the displacement field is related to the object via the phase 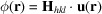 (Robinson & Harder, 2009 ▸). The scalar product **G**_*hkl*_ · **u**(**r**) is the projection of **u**(**r**) onto **G**_*hkl*_ so that the phase in turn is proportional to the displacement field in the direction of the chosen reciprocal space vector. In a ptychographic measurement, the sample is translated in the beam, represented by the complex function 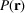 , with overlapping positions *j*, while the scattered intensity is measured at each position. The scattered wave at each position is given by 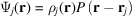 . The propagation to the far field corresponds to a Fourier transform, and the diffraction pattern intensity in three dimensions is 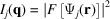 .

However, a detector in the far field samples a 2D slice of the diffraction pattern and not the full 3D intensity. The rotation of the sample determines where it is sliced [see Fig. 1 ▸(*a*)]. Given a complex 3D object and its 3D Fourier transform, it is known from the projection slice theorem that a 2D slice of the 3D Fourier transform is related to a projection of the complex object. To describe a 2D slice of a diffraction pattern in Bragg ptychography and how it depends on the angle, following Hill *et al.* (2018 ▸), we describe deviations from the Laue condition with Δ**q** = **q** − **G**_*hkl*_. A phase factor

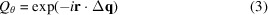

(Hill *et al.*, 2018 ▸; Dzhigaev *et al.*, 2017 ▸; Cha *et al.*, 2016 ▸) is introduced, which allows the intensity to be described using (Hill *et al.*, 2018 ▸)

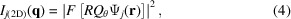

where the projection operator *R* is an integral over the direction of the exit wave **k**_f_. If the 2D slice is measured exactly at the Bragg condition, then Δ**q** = 0 and *Q*_θ_ = 1, and the intensity corresponds to the projected complex density over the direction **k**_f_ (Hruszkewycz *et al.*, 2017 ▸). The interpretation of this projection is straightforward if there is no variation along **k**_f_, but for the general case with variation it is not possible to retrieve the complex density in three dimensions from one angle.

Strain is a local deformation of a solid. To calculate the local strain from BPP, we can use a common definition of strain, which is the derivative of the displacement in the limit of small displacements, *e.g.* 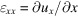 . In scanning XRD, we do not probe the local displacement field but rather the local lattice plane distance. The position of the Bragg peak **G** *_hkl_* at each scanning position is found using the center of mass (COM), and the local lattice plane distance is calculated with equation (1). Then, the strain is calculated relative to some reference value for the lattice plane spacing 〈*d*〉 as

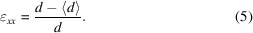

This approach assumes that a local lattice plane distance is well defined within a single beam position, but in general it will vary in three dimensions just like the displacement field. Note also that the relevant volume for this variation is larger than in phase retrieval methods such as BPP, since the spatial resolution is worse. In nanobeam scanning XRD experiments, the beam is often coherent within the illuminated volume. In our case, the highly strained crystal and coherent illumination lead to a complex, asymmetric Bragg peak [see Fig. 1 ▸(*a*)], and it is not obvious that the COM gives an accurate measure of the local lattice plane distance. A challenge in both cases is to define the reference point of the Bragg peak **G**_*hkl*_. One possibility is to use tabulated values for unstrained bulk material, which require precise calibration of the measurement. Another possibility is to use an internal reference point, which means that conclusions can only be drawn about the internal variation.

A key question is how to interpret the projected complex density if the displacement field varies substantially along **k**_f_. In the case of our nanowires, the FEM model indicates that the largest segments have a substantial variation in three dimensions, while the shortest segments are more homogeneous [Fig. 1 ▸(*d*)]. As discussed above, there is no route to retrieve the complex 3D density and therefore the 3D strain. However, the complex density and strain retrieved from BPP can still be used to test a 3D model of the displacement field by comparing the 2D projections.

Here, we compare both approaches with FEM simulations of the nanowire based on the linear theory of elasticity. Bragg peaks were simulated in three dimensions from the FEM model using the beam from the experiment, propagated numerically in the near field to a 3D volume. Finally, simulated diffraction frames were extracted from the 3D Bragg peaks as 2D slices. Thus, the projection is performed as a slice in reciprocal space, not as an integral in real space. These frames were used to perform BPP reconstructions of the simulated nanowire.

### 2.2. Sample

The Ga_*x*_In_1−*x*_P/InP axial heterostructured nanowire studied in this work has five pure InP segments of different sizes, schematized in Fig. 1 ▸(*a*), and is part of a batch of nanowires with an average radius of about 95 nm and about 2.2–2.5 µm length. The structures were grown in the (111)B direction of the zinc blende crystal structure, in particle-assisted growth mode using metal–organic vapor phase epitaxy (Otnes *et al.*, 2016 ▸), and transferred to a Si_3_N_4_ membrane before the experiment. For details on growth parameters, see the supporting information. Previous work has shown a Ga content of around *x* = 21% (Hammarberg *et al.*, 2020 ▸). A gradient of the 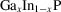 composition has been observed along the nanowire growth axis (Otnes *et al.*, 2017 ▸), with a lower Ga fraction closer to the Au seed particle quantified to 3% per micrometre by Hammarberg *et al.* (2020 ▸). The sample was mounted with the nanowire long axis horizontally oriented, on a piezoelectric stage, translatable on three axes. This enabled probing of the 111 reflections of both GaInP and InP, which are sensitive to the axial strain in the nanowire segments.

### 2.3. Experiment

The X-ray measurements were performed at the MAX IV Laboratory in Lund, Sweden, at the end-station NanoMAX. The photon energy was 10.0 keV and the Kirkpatrick–Baez (KB) mirror setup was employed to focus the X-ray beam. In order to guarantee sufficient overlap in the ptychographic measurements, the beam was enlarged to 180 × 180 nm by reducing the entrance aperture to the KB mirrors (Carbone *et al.*, 2022 ▸). The beam damage on the nanowire was tested for a number of beam intensities before setting the flux to 2.5 × 10^9^ photons s^−1^, at which no beam damage could be detected on the timescale of the experiment. We used an X-ray fluorescence (XRF) detector (silicon drift diode) to locate single nanowires on the substrate. A 2D detector (Merlin QUAD) was positioned in Bragg geometry on a robot arm 1 m from the sample at *2θ* = 21.3° using a helium-filled flight tube.

The horizontal and vertical axes on the 2D detector measured *q*_1_ and *q*_2_, respectively, in the reciprocal space frame (*q*_1_, *q*_2_, *q*_3_), while rotation in θ_B_ along the rocking curve sampled the third axis *q*_3_ [Fig. 1 ▸(*a*)]. The setup was adjusted to fit the diffracted signal from both the InP and the GaInP segments on the Bragg detector. As an example, diffraction summed over a single line *x* at an angle of *θ*_B_ = 10.8° is displayed in Fig. 1 ▸(*a*), with the GaInP peak at a higher *q*_1_, corresponding to a relatively small *d*_111_ spacing.

The dataset was measured with fly scanning (Chahine *et al.*, 2014 ▸) by continuously moving the sample in *x* on a piezoelectric stage, triggering the detector to acquire a frame at a set rate. This reduces the measurement time as the piezo motors do not need to accelerate and settle more than once per row. The sample was scanned with 67 points along the nanowire long axis and 13 points along the radial axis. This yielded approximate steps in *x* and *y* of 50 nm. The acquisition time for the fly scans was set to 0.1 s, giving a total exposure time per 2D Bragg ptychography dataset of only 1.5 min (2.5 min with overhead). This was repeated along the rocking curve, covering the angular range of both GaInP and InP, by rotating the sample in steps of dθ_B_ = 0.02° in 62 steps resulting in a total angular range of 1.24° and a measurement time of about 2.5 h, including overhead time.

### 2.4. Data analysis

In the pre-processing of the diffraction data, the fluorescence signal was used to perform a rough alignment of the data, compensating for real space movements between rotations in θ_B_. Finer alignment was made with intensity maps from the InP diffraction data. After real space alignment, the dataset was reduced to 60 points in *x* and 8 points in *y*, yielding 480 positions, still covering the full nanowire.

The scanning XRD data analysis was achieved by stacking the frames originating from the same real space position, creating a 3D reciprocal space volume. The position of the Bragg peak was found by calculating the COM so that the length of 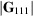 could be calculated. This analysis, used in our previous work (Hammarberg *et al.*, 2020 ▸), gives the relative average strain and the two lattice tilts in the 3D volume illuminated by the beam at each position.

The complex beam profile *P*(**r**) was acquired from transmission ptychography on a Siemens star and propagated to the nanowire sample plane before being used in the BPP reconstructions, where it was kept fixed. The average center and region of interest of scattering on the detector were found for InP and GaInP, respectively. This signal was used to perform BPP reconstructions of the GaInP and InP separately and for every angle θ_B_. Phase retrieval was achieved with the ptychography reconstruction software *Ptypy* (Enders & Thibault, 2016 ▸) with 300 iterations of ePIE (Maiden & Rodenburg, 2009 ▸). The phases were wrapped between [−π, π]. To avoid discontinuities in the phase, it was unwrapped.

We performed FEM simulations in the simulation software COMSOL *Multiphysics*. The assumed lattice mismatch between GaInP and InP in *y* and *z* was 1.52%, as estimated from previous measurements (Hammarberg *et al.*, 2020 ▸). Details about the model can be found in S4 of the supporting information. Furthermore, simulated diffraction frames were calculated from the FEM model using the beam from the experiment, propagated numerically in the near field to a 3D volume; for details, see Hammarberg *et al.* (2020 ▸). These frames were used to perform BPP reconstructions of the simulated nanowire.

## 3. Results and discussion

### 3.1. Comparison of scanning XRD and BPP for the InP segments

The results from the FEM simulation are shown in Fig. 1 ▸(*d*). GaInP has a smaller *d*_111_ spacing relative to InP, exerting compressive stress on the InP in the radial direction, which leads to mostly tensile strain in the measured axial direction. The larger segments are more resilient to the lattice mismatch and show on average lower strain 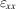 compared with the smaller ones.

**Figure 2:**
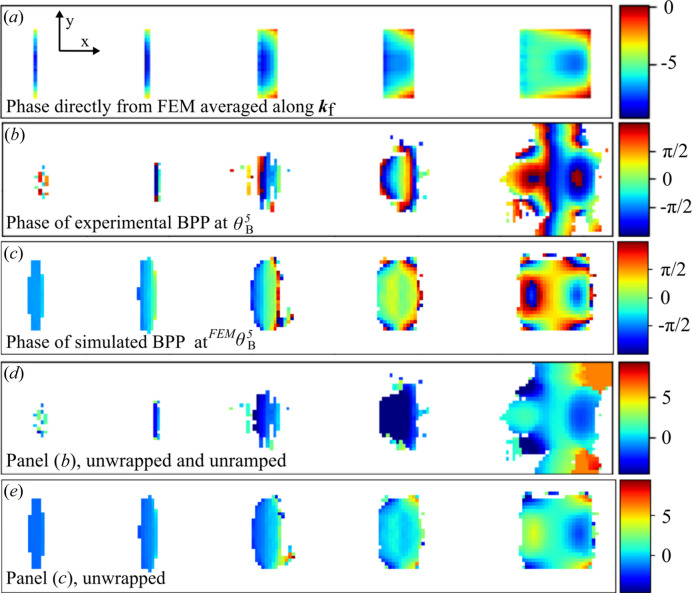
Phase of simulations and BPP reconstructions of the InP segments. (*a*) Displacement field **u**(**r**) from the FEM model averaged along **k**_f_ and converted to phase. (*b*) Phase of the BPP reconstruction at the Bragg condition for segment 5, 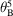 . (*c*) Phase of the simulated BPP reconstruction at the Bragg condition of segment 5, 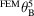 . (*d*) Unramped and unwrapped phase in (*b*). (*e*) Unwrapped phase in (*c*).

The first comparison of the measurements is based on the phases, as shown in Fig. 2 ▸. Fig. 2 ▸(*a*) shows the result from a naïve averaging along **k**_f_ of the displacement field directly from the FEM model, which is then converted to phase. As discussed above, this should only be correct in cases where there is little variation along **k**_f_. The panels below show the phases from the experimental and the simulated BPP. Both the simulated and experimental BPP have phase wraps, as shown in Figs. 2 ▸(*b*) and 2 ▸(*c*), and experimental BPP additionally has phase ramps, which were corrected in Figs. 2 ▸(*d*) and 2 ▸(*e*). The measurement was done at the Bragg angle of segment 5, 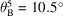 . However, due to the strain differences and a small bending of the nanowire, the Bragg angle was different for the other segments, as shown in Fig. 1 ▸(*c*) and discussed further below.

The BPP reconstruction shown in Fig. 2 ▸(*b*) resolves the inner structure of the largest segments with a pixel size of 8.8 nm. The actual spatial resolution is estimated to be about 40 nm (see Fig. S2), which is an improvement on the 100 nm which is the minimum focus size at 10 keV at NanoMAX. The experimental reconstruction shows a good agreement with both the FEM model and the simulated experiment.

**Figure 3:**
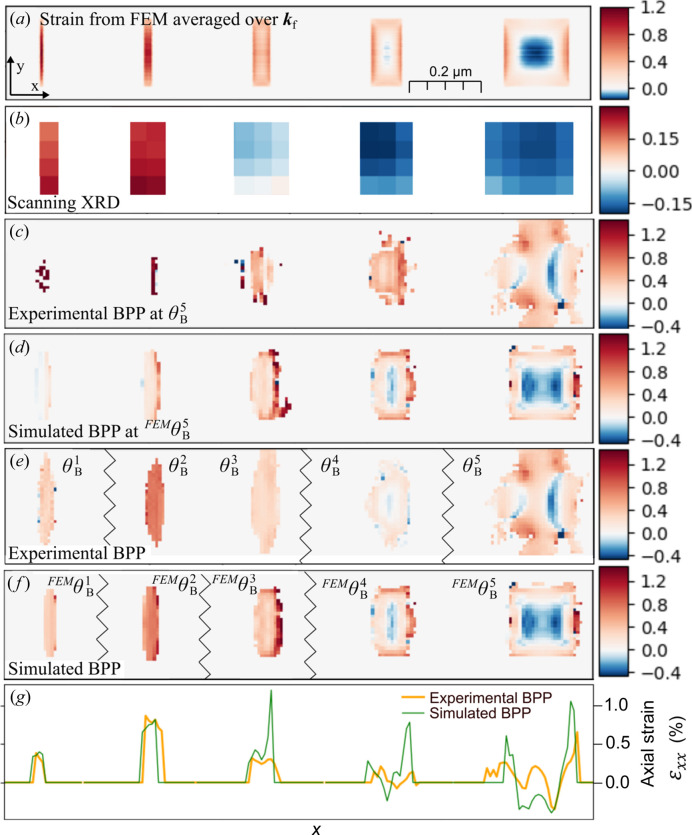
Strain in the InP segments. (*a*) Axial strain 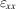 from FEM averaged along **k**_f_. (*b*) Axial strain 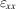 from scanning XRD. Note the deviating color scale. (*c*) Axial strain 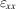 calculated from the phase of the experimental BPP reconstruction at the Bragg angle of segment 5, 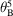 . (*d*) Axial strain 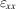 calculated from the phase of the simulated BPP reconstruction at the Bragg angle of segment 5, 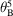 . (*e*) Axial strain 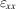 composed from the experimental BPP reconstructions at the respective Bragg angle of each segment 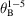 , as displayed in Fig. 1 ▸(*c*). The Bragg angles for segment 2 and segment 3 are the same, 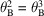 , and hence these are from the same BPP reconstruction. (*f*) Axial strain 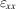 composed from the simulated BPP reconstructions at the respective Bragg angle of each segment 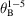 , as displayed in Fig. 1 ▸(*d*). Since the bending of the nanowire is not present in the FEM simulation, the Bragg angles are different from the experimental ones. Here, 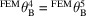 . Note that the color scales in (*a*) and (*c*)–(*f*) are the same, but differ from (*b*). (*g*) Outline of (*e*) and (*f*), at the center of the nanowire. All strains are given as percentages.

Next, we compare the two types of strain that can be calculated from these measurements in Fig. 3 ▸. The first panel, Fig. 3 ▸(*a*), shows the strain calculated from the FEM model and averaged along the **k**_f_ direction. The scanning XRD strain in Fig. 3 ▸(*b*) was calculated directly from the COM of the Bragg peak as discussed above. The COM analysis also generates the two local crystal tilts, which are shown in Fig. S1 of the supporting information. Note that there is an approximately linear gradient in the out-of-plane tilt, β, aside from the local variations. The linear gradient is about dβ/d*x* = −5 mrad µm^−1^, which corresponds to a single radius of curvature of 200 µm (Wallentin *et al.*, 2015 ▸). The reason for this bending is not clear, but it could be related to adhesion to the flexible Si_3_N_4_ membrane.

In contrast, the strain in the BPP simulations and measurements was calculated from the gradient of the displacement fields. Figs. 3 ▸(*c*) and 3(*d*) show the results at the Bragg angle of segment 5, 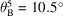 , which gives low-quality reconstructions for the smaller segments. Figs. 3 ▸(*e*) and 3(*f*) therefore show the results for each Bragg angle, as further discussed in the next subsection. Finally, 3 ▸(*g*) shows a lineout comparing the experimental and simulated BPP.

The pixel size in the scanning XRD map, shown in Fig. 3 ▸(*a*), is given by the step size in the measurement, 50 nm, but the resolution is limited by the size of the beam, 180 nm. This is sufficient to resolve the individual segments and variations between them, but not variations within the segments. The NanoMAX beamline is capable of focusing to between 40 and 200 nm, depending on the energy (Carbone *et al.*, 2022 ▸), giving a corresponding improvement in resolution that can be used to image some variation within segments (Hammarberg *et al.*, 2020 ▸). In this experiment, the larger beam was used in order to obtain sufficient overlap for the ptychographic reconstruction.

For the largest segment, the FEM simulation in Fig. 2 ▸(*a*) displays a strain distribution 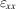 with a sign switch between the edges and the middle. In scanning XRD, this variation is completely blurred. In the experimental BPP in Fig. 3 ▸(*b*), the strain distribution does display a sign switch, but in the form of two lobes of negative strain. The distribution agrees both qualitatively and quantitatively with the BPP simulation in Fig. 3 ▸(*d*), which is not so surprising given that the respective phase maps are similar. The BPP results for the largest segment show quite a bit of qualitative and quantitative similarity with the FEM simulation, despite the averaging effect along the **k**_f_ direction. However, the two lobes do not seem to correspond to a physical 3D strain variation in the 3D FEM model.

A discrepancy between the measured and simulated BPP is a slight asymmetry to the left and right of segment 5, see Figs. 3 ▸(*c*) and 3 ▸(*d*). This could be caused by a slight gradient in the composition of the Ga, discussed in Section 3.3 and also in our previous report (Hammarberg *et al.*, 2020 ▸), which results in a higher lattice mismatch on the right side of segment 5.

**Figure 4:**
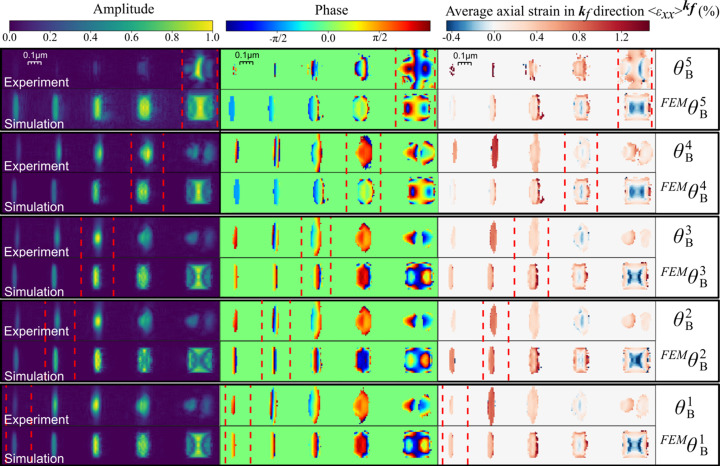
Angular dependence of the BPP reconstructions from simulated and measured diffraction at the diffraction angles of each segments’ Bragg peak denoted 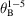 and 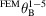 , respectively. Shown here are the reconstructed amplitude, phase and axial strain 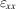 calculated from the unwrapped phase. The phase and strain are masked with a binary mask based on the intensity of the amplitude.

So far, we have discussed the results for segment 5, which is in the Bragg condition in Fig. 3 ▸(*c*). To continue the discussion, it is important to note that segments 1–4 are slightly out of the Bragg condition at this angle 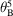 . As visible in Fig. 1 ▸(*c*), the Bragg angles are different for the InP segments because the segments are strained differently, due to their lengths. The angular variation of 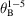 is also affected by a slight bending of the nanowire, shown in Fig. S1, which tilts the lattice. If BPP is performed at an angle away from the Bragg condition, the amplitude and phase reconstruction deviate, which can be seen in Fig. 4 and is discussed further in Section 3.2. To remediate this, we create a composite image where the strain in each of the segments 1–5 is calculated from the reconstruction at its respective Bragg angle 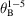 , defined as the angle with highest diffraction intensity in the rocking curve, shown in Fig. 3 ▸(*e*). Since, in this case, the Bragg angles were the same for segments 2 and 3, reconstructions at four angles are shown in Fig. 3 ▸(*e*). Likewise, a simulated BPP experiment, based on the FEM model in Fig. 2 ▸(*a*), is presented for comparison in Fig. 3 ▸(*f*). Note that the angles do not match exactly since the FEM model does not have any bending.

We now compare the smaller segments. In the second largest segment, BPP is able to image some of the internal variation, including a change of sign, which matches the FEM model well both qualitatively and quantitatively. Note that the BPP color scale here is the same as the one for the FEM model, whereas the scanning XRD had to be rescaled to visualize variations. For the smaller segments, BPP does not reveal any internal variation, but the variation between the segments matches well. Note that there is less internal variation in the smaller segments, which makes the averaging effect along the **k**_f_ direction less problematic. An exception is that the smallest segment 1 shows less strain than segment 2 in all maps, in disagreement with FEM. The signal from segment 1 is both weaker than the others and more spread out in reciprocal space, presumably leading to a less reliable reconstruction.

For scanning XRD, the absolute strain is highly dependent on the point of reference, but the differences within and between segments can be compared. We find that, although scanning XRD shows the correct trend with increasing strain for smaller segments, the magnitudes of the differences are much too small.

### 3.2. Angular dependence of Bragg projection ptychography

The InP segments have different Bragg angles, which complicates the BPP measurements and analysis. The strain is different because of the segment lengths, and this, together with a bending of the nanowire, changes the Bragg angle. Measuring BPP off the Bragg condition reduces the scattered signal, which makes the phase retrieval more challenging, and it adds a phase factor as shown in equation (5).

We therefore investigated the effect of measuring BPP at different angles, as shown in Fig. 4 ▸. The reconstructed amplitude, phase and axial strain 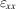 are shown at the respective Bragg angles for the five segments 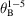 . The experimental maps are shown together with reconstructions of simulated diffraction patterns from the FEM model of the nanowire. The parts of the maps marked with red dashed lines indicate the part of the map that is at the Bragg angle.

The experimental BPP maps vary significantly with angle, affecting amplitude, phase and strain. The amplitude of segment 5 displays internal structure, also at the Bragg condition at 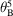 . Away from the Bragg condition, the amplitude is smeared, generally lower and uneven. The simulated BPP is rather robust for small deviations from the Bragg angle, particularly for the largest segment. The strain in the smaller segments shows some variation with angle. These results suggest that the sensitivity to the Bragg alignment is mainly because the lower scattered intensity away from the Bragg condition makes the phase retrieval more challenging.

### 3.3. Bragg projection ptychography of the GaInP segments

**Figure 5:**
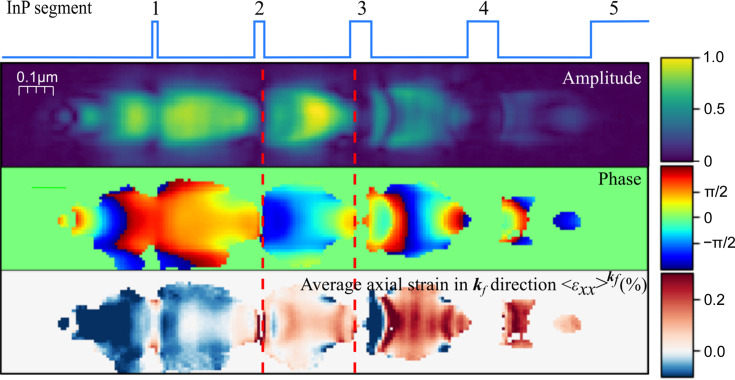
BPP reconstruction from measured diffraction of the GaInP segments at a single angle. The positions of the reconstructed InP segments 1–5 are outlined at the top for orientation. The GaInP segment marked with dashed red lines is in the Bragg condition at this angle, θ_B_ = 11.1°, whereas the other six GaInP segments are out of the Bragg condition.

The results so far have displayed analysis from the InP diffraction, but we also simultaneously measured the GaInP diffraction. A BPP reconstruction of GaInP at 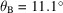 is displayed in Fig. 5 ▸. Five of the six GaInP segments are visible in the reconstruction. Only the segment marked with the red dashed lines is in the Bragg condition.

The quality of the reconstructions is generally much worse than for the InP ones. The strain should have a similar distribution as for InP segment 5 but with the opposite sign [see Fig. 2 ▸(*a*)], but the lobes of negative strain are only vaguely discernible for two of the GaInP segments. The reconstructions are challenged by the fact that the beam is simultaneously illuminating several segments since the InP segments are too small for proper separation. The diffracted signal at a single position will be the coherent sum of the diffraction from two partially illuminated GaInP segments simultaneously. Consequently, the amplitudes of the segments overlap in the reconstructions.

The lengths of the GaInP segments are approximately the same, apart from the right-most one. However, there is an added complexity in composition originating from the growth process. The In is supplied primarily via surface diffusion, and hence, the proportion of In relative to Ga decreases as the nanowire grows longer (Troian *et al.*, 2018 ▸; Berg *et al.*, 2015 ▸). Thus, the ratio *x* in the Ga_*x*_In_1−*x*_P segments increases to the left in our geometry. Therefore, there is a slight gradient in the lattice constant with a smaller lattice constant, and thus higher heterostructure lattice mismatch, towards the seed particle (Hammarberg *et al.*, 2020 ▸; Otnes *et al.*, 2017 ▸). Due to this gradient and the nanowire bending, the Bragg condition is not the same for all GaInP segments. This is observable in the reconstruction as not all segments are reconstructed in amplitude at the same angle.

## 4. Conclusions

We have demonstrated that the complex strain distribution in an axial semiconductor nanowire heterostructure can be probed with BPP. We have compared the phase and strain with simulations and with scanning XRD. As expected, BPP has improved the spatial resolution compared with conventional scanning XRD, revealing the internal structure. The phase maps show good agreement with the simulations.

The FEM model assumes an elastic accommodation of the lattice mismatch without defects. Thus, the quantitative agreement of the FEM model with the ptychographic data suggests that the nanowire could accommodate the mismatch, even though the nanowire is larger than the predicted critical radius (Ertekin *et al.*, 2005 ▸). One reason for this could be that the interfaces are not atomically sharp. Previous transmission electron microscopy (TEM) investigations have not revealed any defects (Hammarberg *et al.*, 2020 ▸), although it is difficult to use TEM to exclude defects.

Overall, we find that the phase and strain calculated from the BPP reconstruction show good qualitative and quantitative agreement with FEM. The exception is the largest segment where we observe clear differences, such as the appearance of two minima. Presumably, this is an effect of the substantial variation of the strain and displacement along ***k***_f_. Meanwhile, the shortest segment is poorly reconstructed, presumably due to the weak signal. However, the middle three segments show excellent agreement for BPP. This can be attributed to the significantly lower internal strain variation in these segments.

Scanning XRD is unable to reveal internal structure, which was expected, but less intuitively we also found that the quantitative differences between segments are too small. A possible explanation for this performance is that the much higher 2D spatial resolution of BPP better captures the variations in displacement in the transverse direction, while scanning XRD averages the entire segment. The transverse spatial resolution of BPP is about 5× better, meaning that the resolved area is on the order of 25× smaller. In our previous work, we performed scanning XRD with a smaller beam (90 nm) which led to a somewhat better quantitative agreement (Hammarberg *et al.*, 2020 ▸). In general, the strain resolution cannot be decoupled from the spatial resolution when there are strong variations. Therefore, phase retrieval methods which improve the real space resolution can also indirectly have better strain resolution.

Although BPP does not provide a 3D view of the strain distribution, we have shown that it can be used to verify a 3D FEM model of the strain at high spatial resolution. BPP is in principle much faster than scanning XRD, not to mention 3D Bragg ptychography, although this is somewhat limited by the demonstrated need to measure each segment at or at least near its Bragg angle.

We have also shown how strain in a heterostructure consisting of several segments of different lengths and strain distributions can be measured with this non-destructive method by constructing a map where each segment is in its Bragg condition. Both types of segments in the heterostructure were probed simultaneously, but internal gradients in composition and lack of separation between the GaInP segments led to a much lower quality reconstruction.

## 5. Related literature

The following additional references are cited in the supporting information: Borgström *et al.* (2010 ▸); Jacobsson *et al.* (2012 ▸).

## Supplementary Material

Supporting figures. DOI: 10.1107/S1600576723010403/xx5037sup1.pdf

## References

[bb1] Barrigón, E., Heurlin, M., Bi, Z., Monemar, B. & Samuelson, L. (2019). *Chem. Rev.* **119**, 9170–9220. 10.1021/acs.chemrev.9b00075 31385696

[bb2] Berenguer, F., Godard, P., Allain, M., Belloir, J. M., Talneau, A., Ravy, S. & Chamard, V. (2013). *Phys. Rev. B*, **88**, 144101.

[bb3] Berg, A., Lenrick, F., Vainorius, N., Beech, J. P., Wallenberg, L. R. & Borgström, M. T. (2015). *Nanotechnology*, **26**, 435601. 10.1088/0957-4484/26/43/435601 26443552

[bb4] Borgström, M. T., Wallentin, J., Trägårdh, J., Ramvall, P., Ek, M., Wallenberg, L. R., Samuelson, L. & Deppert, K. (2010). *Nano Res.* **3**, 264–270.

[bb5] Carbone, D., Kalbfleisch, S., Johansson, U., Björling, A., Kahnt, M., Sala, S., Stankevic, T., Rodriguez-Fernandez, A., Bring, B., Matej, Z., Bell, P., Erb, D., Hardion, V., Weninger, C., Al-Sallami, H., Lidon-Simon, J., Carlson, S., Jerrebo, A., Norsk Jensen, B., Bjermo, A., Åhnberg, K. & Roslund, L. (2022). *J. Synchrotron Rad.* **29**, 876–887. 10.1107/S1600577522001333 PMC9070697 35511021

[bb6] Cha, W., Ulvestad, A., Allain, M., Chamard, V., Harder, R., Leake, S. J., Maser, J., Fuoss, P. H. & Hruszkewycz, S. O. (2016). *Phys. Rev. Lett.* **117**, 225501. 10.1103/PhysRevLett.117.225501 27925753

[bb101] Chahine, G. A., Richard, M.-I., Homs-Regojo, R. A., Tran-Caliste, T. N., Carbone, D., Jacques, V. L. R., Grifone, R., Boesecke, P., Katzer, J., Costina, I., Djazouli, H., Schroeder, T. & Schülli, T. U. (2014). *J. Appl. Cryst.* **47**, 762–769.

[bb7] Chamard, V., Allain, M., Godard, P., Talneau, A., Patriarche, G. & Burghammer, M. (2015). *Sci. Rep.* **5**, 9827. 10.1038/srep09827 PMC4434906 25984829

[bb8] Diaz, A., Mocuta, C., Stangl, J., Mandl, B., David, C., Vila-Comamala, J., Chamard, V., Metzger, T. H. & Bauer, G. (2009). *Phys. Rev. B*, **79**, 125324.

[bb9] Dzhigaev, D., Shabalin, A., Stankevič, T., Lorenz, U., Kurta, R. P., Seiboth, F., Wallentin, J., Singer, A., Lazarev, S., Yefanov, O. M., Borgström, M., Strikhanov, M. N., Samuelson, L., Falkenberg, G., Schroer, C. G., Mikkelsen, A., Feidenhans’l, R. & Vartanyants, I. A. (2016). *J. Opt.* **18**, 064007.

[bb10] Dzhigaev, D., Stankevič, T., Bi, Z., Lazarev, S., Rose, M., Shabalin, A., Reinhardt, J., Mikkelsen, A., Samuelson, L., Falkenberg, G., Feidenhans’l, R. & Vartanyants, I. A. (2017). *ACS Nano*, **11**, 6605–6611. 10.1021/acsnano.6b08122 28264155

[bb11] Dzhigaev, D., Svensson, J., Krishnaraja, A., Zhu, Z., Ren, Z., Liu, Y., Kalbfleisch, S., Björling, A., Lenrick, F. & Balogh, Z. I. (2020). *Nanoscale*, **12**, 14487–14493. 10.1039/d0nr02260h 32530025

[bb12] Enders, B. & Thibault, P. (2016). *Proc. Math. Phys. Eng. Sci.* **472**, 20160640. 10.1098/rspa.2016.0640 PMC5247528 28119552

[bb13] Ertekin, E., Greaney, P. A., Chrzan, D. C. & Sands, T. D. (2005). *J. Appl. Phys.* **97**, 114325.

[bb14] Fienup, J. R. (1982). *Appl. Opt.* **21**, 2758–2769. 10.1364/AO.21.002758 20396114

[bb15] Gibson, S. J., van Kasteren, B., Tekcan, B., Cui, Y., van Dam, D., Haverkort, J. E., Bakkers, E. P. & Reimer, M. E. (2019). *Nat. Nanotechnol.* **14**, 473–479. 10.1038/s41565-019-0393-2 30833690

[bb16] Godard, P., Carbone, G., Allain, M., Mastropietro, F., Chen, G., Capello, L., Diaz, A., Metzger, T. H., Stangl, J. & Chamard, V. (2011). *Nat. Commun.* **2**, 568. 10.1038/ncomms1569 22127064

[bb17] Gudiksen, M. S., Lauhon, L. J., Wang, J., Smith, D. C. & Lieber, C. M. (2002). *Nature*, **415**, 617–620. 10.1038/415617a 11832939

[bb18] Hammarberg, S., Dagytė, V., Chayanun, L., Hill, M. O., Wyke, A., Björling, A., Johansson, U., Kalbfleisch, S., Heurlin, M., Lauhon, L. J., Borgström, M. T. & Wallentin, J. (2020). *Nano Res.* **13**, 2460–2468.

[bb19] Haverkort, J. E. M., Garnett, E. C. & Bakkers, E. P. A. M. (2018). *Appl. Phys. Rev.* **5**, 031106.

[bb20] Hill, M. O., Calvo-Almazan, I., Allain, M., Holt, M. V., Ulvestad, A., Treu, J., Koblmüller, G., Huang, C., Huang, X., Yan, H., Nazaretski, E., Chu, Y. S., Stephenson, G. B., Chamard, V., Lauhon, L. J. & Hruszkewycz, S. O. (2018). *Nano Lett.* **18**, 811–819. 10.1021/acs.nanolett.7b04024 29345956

[bb21] Holt, M. V., Hruszkewycz, S. O., Murray, C. E., Holt, J. R., Paskiewicz, D. M. & Fuoss, P. H. (2014). *Phys. Rev. Lett.* **112**, 165502. 10.1103/PhysRevLett.112.165502 24815657

[bb22] Hrachowina, L., Chen, Y., Barrigon, E., Wallenberg, R. & Borgström, M. T. (2022). *Mater. Today Energy*, **27**, 101050.

[bb24] Hruszkewycz, S. O., Allain, M., Holt, M. V., Murray, C. E., Holt, J. R., Fuoss, P. H. & Chamard, V. (2017). *Nat. Mater.* **16**, 244–251. 10.1038/nmat4798 27869823

[bb25] Hruszkewycz, S. O., Holt, M. V., Murray, C. E., Bruley, J., Holt, J., Tripathi, A., Shpyrko, O. G., McNulty, I., Highland, M. J. & Fuoss, P. H. (2012). *Nano Lett.* **12**, 5148–5154. 10.1021/nl303201w 22998744

[bb26] Huang, X., Harder, R., Leake, S., Clark, J. & Robinson, I. (2012). *J. Appl. Cryst.* **45**, 778–784. 10.1107/S0021889812018900 PMC3401992 22829708

[bb28] Hÿtch, M. J. & Minor, A. M. (2014). *MRS Bull.* **39**, 138–146.

[bb29] Jacobsson, D., Persson, J. M., Kriegner, D., Etzelstorfer, T., Wallentin, J., Wagner, J. B., Stangl, J., Samuelson, L., Deppert, K. & Borgström, M. T. (2012). *Nanotechnology*, **23**, 245601. 10.1088/0957-4484/23/24/245601 22641029

[bb30] Jia, C., Lin, Z., Huang, Y. & Duan, X. (2019). *Chem. Rev.* **119**, 9074–9135. 10.1021/acs.chemrev.9b00164 31361471

[bb31] Krause, T., Hanke, M., Cheng, Z., Niehle, M., Trampert, A., Rosenthal, M., Burghammer, M., Ledig, J., Hartmann, J., Zhou, H., Wehmann, H.-H. & Waag, A. (2016). *Nanotechnology*, **27**, 325707. 10.1088/0957-4484/27/32/325707 27352816

[bb32] LaPierre, R. R., Chia, A. C. E., Gibson, S. J., Haapamaki, C. M., Boulanger, J., Yee, R., Kuyanov, P., Zhang, J., Tajik, N., Jewell, N. & Rahman, K. M. A. (2013). *Phys. Status Solidi Rapid Res. Lett.* **7**, 815–830.

[bb33] Lazarev, S., Dzhigaev, D., Bi, Z., Nowzari, A., Kim, Y. Y., Rose, M., Zaluzhnyy, I. A., Gorobtsov, O. Y., Zozulya, A. V., Lenrick, F., Gustafsson, A., Mikkelsen, A., Sprung, M., Samuelson, L. & Vartanyants, I. A. (2018). *Nano Lett.* **18**, 5446–5452. 10.1021/acs.nanolett.8b01802 30033733

[bb34] Li, P., Allain, M., Grünewald, T. A., Rommel, M., Campos, A., Carbone, D. & Chamard, V. (2022). *Light Sci. Appl.* **11**, 73. 10.1038/s41377-022-00758-z PMC8956681 35338112

[bb35] Li, P., Phillips, N. W., Leake, S., Allain, M., Hofmann, F. & Chamard, V. (2021). *Nat. Commun.* **12**, 7059. 10.1038/s41467-021-27224-5 PMC8642407 34862390

[bb36] Maiden, A. M. & Rodenburg, J. M. (2009). *Ultramicroscopy*, **109**, 1256–1262. 10.1016/j.ultramic.2009.05.012 19541420

[bb37] Marçal, L. A. B., Oksenberg, E., Dzhigaev, D., Hammarberg, S., Rothman, A., Björling, A., Unger, E., Mikkelsen, A., Joselevich, E. & Wallentin, J. (2020). *ACS Nano*, **14**, 15973–15982. 10.1021/acsnano.0c07426 PMC7690043 33074668

[bb38] Marçal, L. A. B., Richard, M.-I., Magalhães-Paniago, R., Cavallo, F., Lagally, M. G., Schmidt, O. G., Schülli, T. Ü., Deneke, C. & Malachias, A. (2015). *Appl. Phys. Lett.* **106**, 151905.

[bb39] Memisevic, E., Hellenbrand, M., Lind, E., Persson, A. R., Sant, S., Schenk, A., Svensson, J., Wallenberg, R. & Wernersson, L.-E. (2017). *Nano Lett.* **17**, 4373–4380. 10.1021/acs.nanolett.7b01455 28613894

[bb40] Miao, J., Charalambous, P., Kirz, J. & Sayre, D. (1999). *Nature*, **400**, 342–344.

[bb41] Motohisa, J., Kameda, H., Sasaki, M. & Tomioka, K. (2019). *Nanotechnology*, **30**, 134002. 10.1088/1361-6528/aafce5 30625458

[bb42] Newton, M. C., Leake, S. J., Harder, R. & Robinson, I. K. (2010). *Nat. Mater.* **9**, 120–124. 10.1038/nmat2607 20023632

[bb43] Otnes, G. & Borgström, M. T. (2017). *Nano Today*, **12**, 31–45.

[bb44] Otnes, G., Heurlin, M., Graczyk, M., Wallentin, J., Jacobsson, D., Berg, A., Maximov, I. & Borgström, M. T. (2016). *Nano Res.* **9**, 2852–2861.

[bb45] Otnes, G., Heurlin, M., Zeng, X. & Borgström, M. T. (2017). *Nano Lett.* **17**, 702–707. 10.1021/acs.nanolett.6b03795 28054783

[bb46] Pateras, A. I., Allain, M., Godard, P., Largeau, L., Patriarche, G., Talneau, A., Pantzas, K., Burghammer, M., Minkevich, A. A. & Chamard, V. (2015). *Phys. Rev. B*, **92**, 205305. 10.1038/srep09827 PMC4434906 25984829

[bb47] Pfeifer, M. A., Williams, G. J., Vartanyants, I. A., Harder, R. & Robinson, I. K. (2006). *Nature*, **442**, 63–66. 10.1038/nature04867 16823449

[bb48] Robinson, I. & Harder, R. (2009). *Nat. Mater.* **8**, 291–298. 10.1038/nmat2400 19308088

[bb49] Rodenburg, J. M., Hurst, A. C., Cullis, A. G., Dobson, B. R., Pfeiffer, F., Bunk, O., David, C., Jefimovs, K. & Johnson, I. (2007). *Phys. Rev. Lett.* **98**, 034801. 10.1103/PhysRevLett.98.034801 17358687

[bb50] Saxena, D., Mokkapati, S., Parkinson, P., Jiang, N., Gao, Q., Tan, H. H. & Jagadish, C. (2013). *Nat. Photon.* **7**, 963–968.

[bb51] Sayre, D. (1952). *Acta Cryst.* **5**, 843.

[bb52] Schülli, T. U. & Leake, S. J. (2018). *Curr. Opin. Solid State Mater. Sci.* **22**, 188–201.

[bb53] Shapiro, D., Thibault, P., Beetz, T., Elser, V., Howells, M., Jacobsen, C., Kirz, J., Lima, E., Miao, H., Neiman, A. M. & Sayre, D. (2005). *Proc. Natl Acad. Sci. USA*, **102**, 15343–15346. 10.1073/pnas.0503305102 PMC1250270 16219701

[bb102] Spolenak, R., Brown, W., Tamura, N., MacDowell, A., Celestre, R., Padmore, H., Valek, B., Bravman, J., Marieb, T., Fujimoto, H. J., Batterman, B. W. & Patel, J. R. (2003). *Phys. Rev. Lett.* **90**, 096102. 10.1103/PhysRevLett.90.096102 12689241

[bb54] Stankevič, T., Hilner, E., Seiboth, F., Ciechonski, R., Vescovi, G., Kryliouk, O., Johansson, U., Samuelson, L., Wellenreuther, G., Falkenberg, G., Feidenhans’l, R. & Mikkelsen, A. (2015). *ACS Nano*, **9**, 6978–6984. 10.1021/acsnano.5b01291 26090689

[bb55] Thibault, P., Dierolf, M., Menzel, A., Bunk, O., David, C. & Pfeiffer, F. (2008). *Science*, **321**, 379–382. 10.1126/science.1158573 18635796

[bb56] Tomioka, K., Yoshimura, M. & Fukui, T. (2012). *Nature*, **488**, 189–192. 10.1038/nature11293 22854778

[bb57] Troian, A., Otnes, G., Zeng, X., Chayanun, L., Dagytė, V., Hammarberg, S., Salomon, D., Timm, R., Mikkelsen, A., Borgström, M. T. & Wallentin, J. (2018). *Nano Lett.* **18**, 6461–6468. 10.1021/acs.nanolett.8b02957 30185034

[bb58] Wallentin, J., Jacobsson, D., Osterhoff, M., Borgström, M. T. & Salditt, T. (2017). *Nano Lett.* **17**, 4143–4150. 10.1021/acs.nanolett.7b00918 28613907

[bb59] Wallentin, J., Osterhoff, M. & Salditt, T. (2016). *Adv. Mater.* **28**, 1788–1792. 10.1002/adma.201504188 26689602

[bb60] Wallentin, J., Wilke, R. N., Osterhoff, M. & Salditt, T. (2015). *J. Appl. Cryst.* **48**, 1818–1826. 10.1107/S1600576715017975 PMC4665660 26664342

[bb61] Yao, M., Cong, S., Arab, S., Huang, N., Povinelli, M. L., Cronin, S. B., Dapkus, P. D. & Zhou, C. (2015). *Nano Lett.* **15**, 7217–7224. 10.1021/acs.nanolett.5b03890 26502060

[bb62] Zeng, X., Otnes, G., Heurlin, M., Mourão, R. T. & Borgström, M. T. (2018). *Nano Res.* **11**, 2523–2531.

