# Supplementary material for: Fast nanoscale imaging of strain in a multi-segment heterostructured nanowire with 2D Bragg ptychography
Source: J Appl Crystallogr. 2024 Feb 1;57(Pt 1):60–70. doi: 10.1107/S1600576723010403 (PMC10840305; doi:10.1107/S1600576723010403)
Supplement: Supplementary file 1 [file j-57-00060-sup1.pdf]

JOURNAL OF APPLIED CRYSTALLOGRAPHY

Volume 57 (2024)

Supporting information for article:

Fast nanoscale imaging of strain in a multi-segment heterostructured nanowire with 2D Bragg ptychography

Susanna Hammarberg, Dmitry Dzhigaev, Lucas Marcal, Vilgaile Dagyte, Alexander Björling, Magnus Borgström and Jesper Wallentin

S1. Growth

The Au seed particle array was defined on a 2” InP (111)B wafer by nanoimprint lithography, reactive ion etching, metal evaporation, and lift-off, resulting in a hexagonal pattern of Au particles with a pitch of 500 nm (Otnes et al., 2016). The imprinted InP wafer was cleaved into smaller samples used for growth.

The nanowires were grown in a low pressure (100 mbar) metal-organic vapor phase epitaxy system (Aixtron 200/4) with a total flow of 13 l/min and H_2 as the carrier gas. In order to improve pattern preservation, a pre-anneal nucleation step (Otnes *et al.*, 2016) was performed at 280 °C for 1 min with molar fractions of trimethylindium (TMIn) of $\chi_{TMIn} = 8.9 \times 10^{-5}$ and phosphine (PH_3) of $\chi_{PH3} = 6.9 \times 10^{-3}$. Then the sample was annealed for 10 min at 550 °C under $\chi_{PH3} = 3.5 \times 10^{-2}$ to desorb surface oxides. After annealing, the chamber was cooled to 440 °C. The growth was initiated with a InP nucleation step by adjusting PH_3 to $\chi_{PH3} = 6.9 \times 10^{-3}$ and introducing TMIn with $\chi_{TMIn} = 8.9 \times 10^{-5}$. After 15 s, hydrogen chloride (HCl) was introduced at a molar fraction of $\chi_{HCl} = 4.6 \times 10^{-5}$ to eliminate radial growth (Borgström *et al.*, 2010; Jacobsson *et al.*, 2012). After a total of 60 s InP growth, 4 min of InGaP growth was carried out by introducing trimethylgallium (TMGa) at a molar fraction of $\chi_{TMGa} = 1.4 \times 10^{-3}$ and by switching the other precursors to $\chi_{TMIn} = 2.7 \times 10^{-5}$, $\chi_{PH3} = 5.4 \times 10^{-3}$, and $\chi_{HCl} = 5.4 \times 10^{-5}$. After this, a barcode sequence was grown by alternating between InP, $\chi_{TMIn} = 5.4 \times 10^{-5}$, and InGaP, $\chi_{TMIn} = 2.7 \times 10^{-5}$ and $\chi_{TMGa} = 1.4 \times 10^{-3}$. PH_3 and HCl were kept constant as in the previous step. The 10 segments were grown each for 2 min, 2 min 50 s, 1 min, 3 min, 45 s, 3 min 10 s, 30 s, 3 min 20 s, 15 s, and 3 min 41 s respectively. After the barcode sequence, TMIn was switched off for 2 min, while keeping PH_3, HCl, and TMGa unchanged. Finally, the flows of TMGa and HCl were switched off and the chamber was cooled to 300 °C under a PH_3/H_2 gas mixture.

S2. Scanning XRD full result

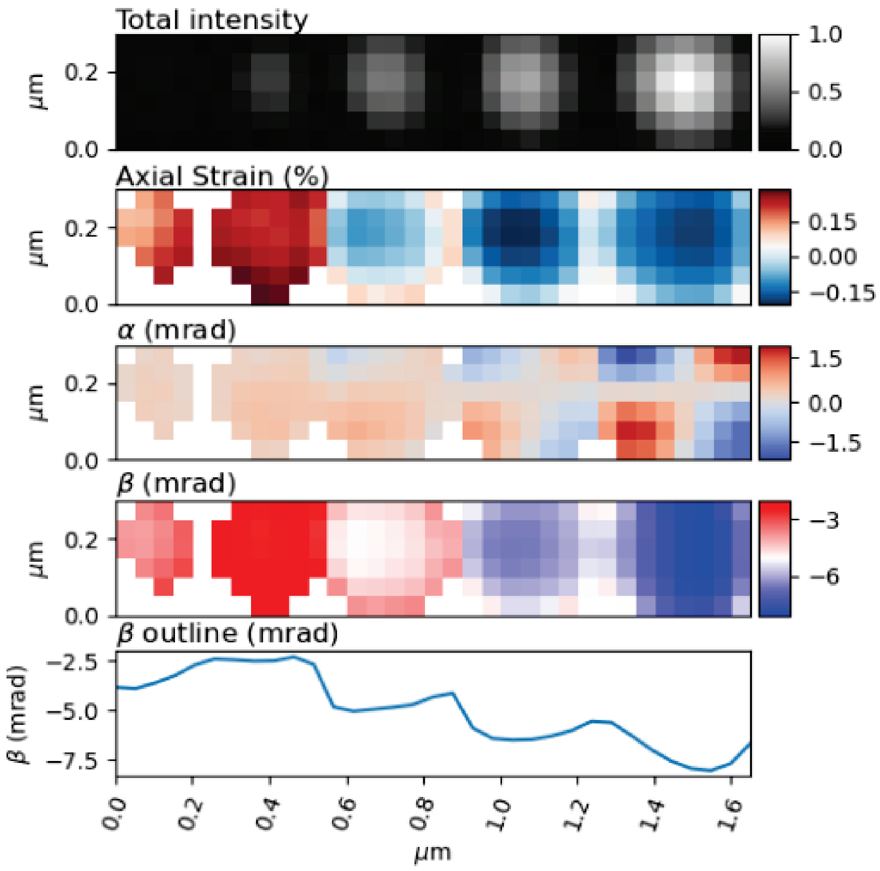

Figure S1 Strain and tilt maps from scanning XRD. Pixel size is 50 x 50 nm. From top: the total intensity from the scattered beam, the axial strain ε_{xx}, the tilt around z, α, tilt around y, β, outline of β at the center of the nanowire, $y = 0.2$.

S3. Resolution estimate 2D Bragg ptychography

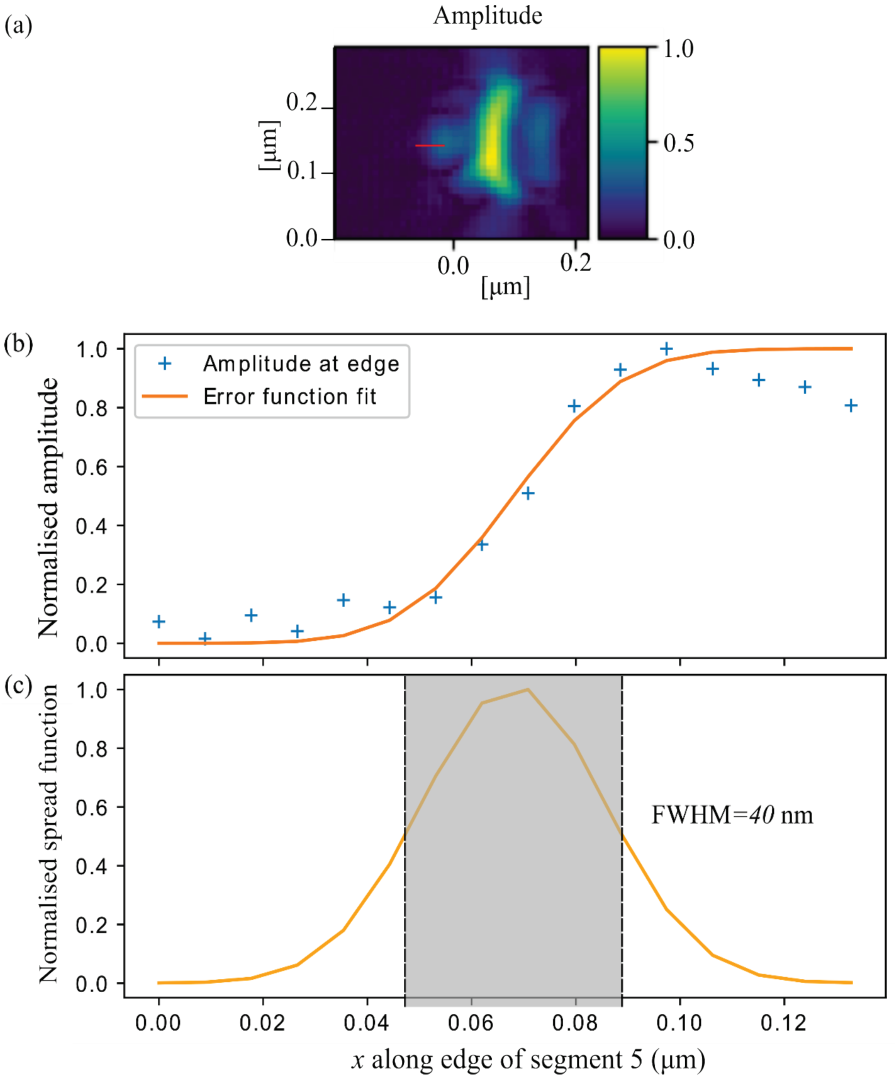

Figure S2 Resolution estimate of the 2D Bragg ptychography reconstructions. (a) Cutout of segment 5 from the reconstructed amplitude from 2D Bragg ptychography at the angle of segment 5. (b) A lineout from the amplitude edge indicated with red line in (a), fitted with an error function. (c) The derivative of the error function in (b), giving the line spread function. The full-width half maximum indicates a resolution of 40 nm.

S4. FEM simulation details

The FEM simulation was performed using an isotropic linear elasticity model in the simulation software COMSOL Multiphysics. We used bulk literature values for the InP material parameters. For $Ga_xIn_{1-x}P$, we used interpolated values between InP and GaP using Vegard's law, assuming x = 21%. From the growth, a 10 nm stub of InP is left on the nanowire. This stub was included model but not shown in the figures. The model was constrained to have the end with the stub fixed. The mesh was constructed with smaller domains closer to the heterostructure interfaces, where strains gradients are high, than in their centers. Typical elements were about one nanometer.

S5. References

Borgström, M. T., Wallentin, J., Trägårdh, J., Ramvall, P., Ek, M., Wallenberg, L. R., Samuelson, L. & Deppert, K. (2010). Nano Res. 3, 264-270.

Jacobsson, D., Persson, J. M., Kriegner, D., Etzelstorfer, T., Wallentin, J., Wagner, J. B., Stangl, J., Samuelson, L., Deppert, K. & Borgström, M. T. (2012). Nanotechnology 23, 245601.

Otnes, G., Heurlin, M., Graczyk, M., Wallentin, J., Jacobsson, D., Berg, A., Maximov, I. & Borgström, M. T. (2016). Nano Research 9, 2852-2861.

Chahine, G. A., Richard, M.-I., Homs-Regojo, R. A., Tran-Caliste, T. N., Carbone, D., Jacques, V. L. R., Grifone, R., Boesecke, P., Katzer, J. & Costina, I. J. J. o. A. C. (2014). **47**, 762-769.

Spolenak, R., Brown, W., Tamura, N., MacDowell, A., Celestre, R., Padmore, H., Valek, B., Bravman, J., Marieb, T. & Fujimoto, H. J. P. r. l. (2003). **90**, 096102.
